# About a rare tumor of the upper lip: the mucoepidermoid carcinoma

**DOI:** 10.11604/pamj.2015.22.39.7177

**Published:** 2015-09-17

**Authors:** Mohamed Mliha Touati, Mohammed Lakouichmi

**Affiliations:** 1 ENT Department, Military Hospital Avicenna, Marrakech, Morocco; 2 Stomatology and Maxillofacial Surgery Department, Military Hospital Avicenna, Marrakech, Morocco

**Keywords:** Minor salivary gland, mucoepidermoid carcinoma, upper lip

## Image in medicine

Mucoepidermoid carcinoma represents about 5% of all salivary gland tumors, in which, about one-third occurs in the minor salivary gland. Mucoepidermoid carcinoma is the third most frequently encountered minor salivary gland tumor, preceded only by a mixed tumor and adenoid cystic carcinoma. The affected minor salivary glands are most frequently located in the palate, followed by the lower lip. Tumors of the minor salivary glands occur predominantly in women in the fourth to fifth decades of life. The tumors are characterized by endophytic growth and often present a slowly progressive course, which delays the diagnosis. We report a 42-year-old woman, who consulted for a painless mass with a diameter of 3x2, 5 cm on the internal aspect of the upper lip. The mass had appeared 3 years earlier. The lesion was lobulated, of elastic consistency, and covered by a normal mucosa. Its growth had been slowly progressive. There were no palpable locoregional lymph nodes. As the swelling was progressively increasing in size, surgical excision of the swelling was planned. The mass was removed under general anesthesia and sent to the Pathology Department for histopathological examination. Low-grade mucoepidermoid carcinoma was diagnosed. Although malignant minor salivary gland tumors are rare in the lip, they must be kept in mind in the differential diagnosis. As the prognosis of low-grade mucoepidermoid carcinoma is excellent after wide local resection, although rare, the possibility of mucoepidermoid carcinoma should be always be considered while dealing with any lip mass.

**Figure 1:**
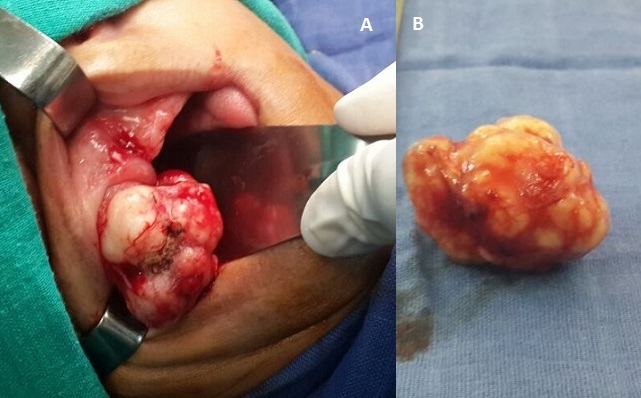
(A) intraoperative, an encapsulated and lobulated tumor of the upper lip; (B) the tumor after surgical resection

